# An investigation into the impact of temporality on COVID-19 infection and mortality predictions: new perspective based on Shapley Values

**DOI:** 10.1186/s12874-025-02572-8

**Published:** 2025-04-24

**Authors:** Mingming Chen, Qihang Qian, Xiang Pan, Tenglong Li

**Affiliations:** 1 Academy of Pharmacy, Xi’an Jiaotong-Liverpool University, 111 Ren’ai Road, Suzhou, 215123 Jiangsu P.R. China; 2 School of Computer Science and Technology, Zhejiang University of Technology, No. 18 Chaowang Road, Hangzhou, Zhejiang 310014 P.R. China; 3 Institute of Population Health, Faculty of Health & Life Sciences Waterhouse Building, University of Liverpool, Liverpool, England

**Keywords:** Temporality import, Shapley values, Random forest, COVID-19 infection prediction, COVID-19 mortality prediction

## Abstract

**Introduction:**

Machine learning models have been employed to predict COVID-19 infections and mortality, but many models were built on training and testing sets from different periods. The purpose of this study is to investigate the impact of temporality, i.e., the temporal gap between training and testing sets, on model performances for predicting COVID-19 infections and mortality. Furthermore, this study seeks to understand the causes of the impact of temporality.

**Methods:**

This study used a COVID-19 surveillance dataset collected from Brazil in year 2020, 2021 and 2022, and built prediction models for COVID-19 infections and mortality using random forest and logistic regression, with 20 model features. Models were trained and tested based on data from different years and the same year as well, to examine the impact of temporality. To further explain the impact of temporality and its driving factors, Shapley values are employed to quantify individual contributions to model predictions.

**Results:**

For the infection model, we found that the temporal gap had a negative impact on prediction accuracy. On average, the loss in accuracy was 0.0256 for logistic regression and 0.0436 for random forest when there was a temporal gap between the training and testing sets. For the mortality model, the loss in accuracy was 0.0144 for logistic regression and 0.0098 for random forest, which means the impact of temporality was not as strong as in the infection model. Shapley values uncovered the reason behind such differences between the infection and mortality models.

**Conclusions:**

Our study confirmed the negative impact of temporality on model performance for predicting COVID-19 infections, but it did not find such negative impact of temporality for predicting COVID-19 mortality. Shapley value revealed that there was a fixed set of four features that made predominant contributions for the mortality model across data in three years (2020–2022), while for the infection model there was no such fixed set of features across different years.

**Supplementary Information:**

The online version contains supplementary material available at 10.1186/s12874-025-02572-8.

## Introduction

The World Health Organization (WHO) officially declared the end of COVID-19 public health emergency, admitting that the world should move forward with uncountable and irreparable scars left by the pandemic. [1] More than 773 million cases have been reported to WHO and the pandemic has claimed millions of lives around the world, with a mortality rate between two to three%.[2] It Is also worth noting that the different variants of SARS-CoV-2, which exhibited distinct epidemiological features and symptoms, have emerged and became dominant at different stages during the pandemic. [3] The Alpha variant (B.1.1.7) was first discovered in the United Kingdom in September 2020. The virus is characterized by high fever [4] and makes older people more vulnerable. The Delta variant (B.1.617.2) was first discovered in India in late 2020. This virus is more likely to cause respiratory symptoms, [5] such as coughing, as well as higher rates of hospitalization and mortality. The Delta variant also exhibited significantly higher transmissibility compared to previous strains. The Omicron variant (B.1.1.529) was first identified in South Africa in November 2021. This variant has attracted concern because of its large number of mutations, including several significant changes to the spike protein on the surface of the virus. Omicron typically causes less severe symptoms, appears to be influenza like illness (ILI) rather than pneumonia, and is less deadly than the previous SARS-CoV-2 variants. [6–7] However, its transmission is also unprecedentedly fast around the world.

The coronavirus was transmitted at a truly astounding speed, but what is more astounding is how it triggers fundamental changes in public health in a digitalized world. Numerous models and software tools have been created to provide support in surveillance, forecasting and policy-making, by collecting and mining data from various sources. [8] A particular worthwhile application is building prediction models for COVID-19 infection and mortality, and literature shows that some models can achieve reasonable accuracies for predicting COVID-19 infection and mortality based on individual features. For example, spatio-temporal modeling approaches using artificial neural network algorithms have been applied to predict COVID-19 prevalence and mortality [9], and GIS-based spatio-temporal analysis has been utilized to model COVID-19 incidence rates in Europe [10]. we can use machine learning algorithms to predict mortality in COVID-19 patients using individual features like chest computed tomography severity scores (CT-SS), demographics, and clinical presentations. [11] Another exemplifying application involves a novel stacking-ensemble model which was designed to predict the total number of COVID-19 patients in 10 Brazilian states for the upcoming 1–3 and 6 days. [12] This innovative model achieved high accuracy of short-term predictions and was employed in an early warning system for guiding healthcare experts and government authorities.

Nonetheless, literature has suggested that machine learning models are largely limited in their robustness and interpretabilities, particularly for prediction tasks related to COVID-19. [13–14] For example, some studies suggested that the elderly and individuals with pre-existing conditions such as diabetes, obesity, and cardiovascular disease had higher infection and mortality rates. [15] Contradictorily, some other studies that were built on different datasets reported high infection and mortality rates among young population and individuals without pre-existing conditions, which indicates many models may not be as robust as we expected. As a result, inconsistencies in terms of model accuracy have been documented even based on similar set of features, [14] and the gap between model for COVID-19 infection and model for COVID-19 mortality is especially notable. [13, 16] Furthermore, confounding factors such as temporality also potentially compromise the performance of machine learning model. As discussed above, the transmission dynamics of COVID-19 has been driven by different variants at different times. Due to the epidemiological distinctions among the variants, a machine learning model built on data collected in year 2020 (when the alpha variant was dominant) is expected to have lower accuracy for data collected in year 2022 (when the Omicron variant was dominant). However, the existing literature does not offer an easily interpretable analysis of the reasons behind the inconsistencies of predictive performances for a fixed model in different contexts. For example, one needs to understand why predictive performance changes based on the same set of features for modeling infections versus deaths or modeling infections across different years. Such interpretable analysis typically demands a clear interpretable quantification of individual feature contributions for a predictive model, to explain the discrepancies in its performances in different contexts.

Bearing the above issue in mind, we focus on evaluating individual feature contributions for models targeting different clinical outcomes of COVID-19 (i.e., infection versus mortality) and models using data in different years, with a commonly-adopted highly-interpretable machine learning index, i.e., Shapley value. Our first goal is to investigate the temporal impact on the model performance for COVID-19 infection, i.e., whether model performance would be compromised by using data from different stages of the pandemic. For example, we are curious that if a model would underperform using a training set from Year 2020 but a testing set from Year 2021 (or 2022). Individual feature contributions would be assessed with Shapley values for models built on different years of data, if inconsistencies regarding model performances indeed exist. Similarly, our second goal is to study the temporal impact on the model performance for COVID-19 mortality, by comparing the model performances and feature contributions using data from different years. Lastly, our third goal is to make a general comparison between the models for COVID-19 infection and mortality, to find out if substantial difference does exist between the two kinds of prediction tasks, and if yes the possible explanations for such difference. By providing a detailed analysis of the impact of temporal factors on the performance of machine learning models for COVID-19 infection and mortality with the aid of Shapley values, our study seeks to understand why prediction accuracy for COVID-19 varies across different stages of the pandemic and interpret the underlying reasons from an epidemiological perspective, based on a large surveillance data in Brazil from 2020 to 2022. Our paper is structured as follows: The method section outlines our modeling workflow and provides the theoretical background of Shapley value. The results section presents the results regarding the models for COVID-19 infection and mortality, as well as a brief summary. The discussion section reviews our main findings from the results as well as potential limitations, along with necessary epidemiological interpretations. The paper is ended with a conclusion section.

## Methods

### Data

**Fig. 1:**
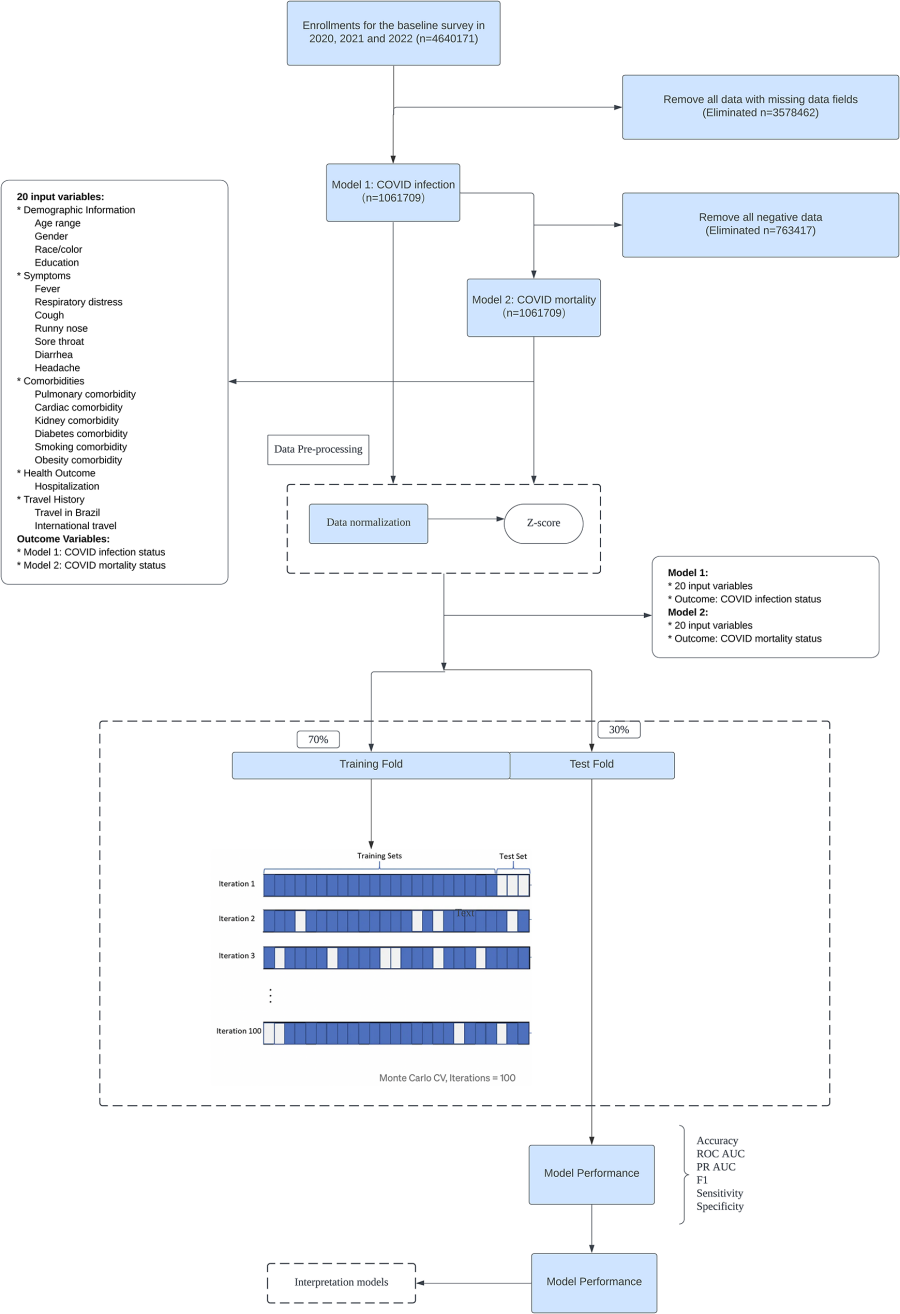
Flowchart of our work in data preprocessing and model building with the Employed MCCV Methodology in this Study

The database used in our study is from the State of Espírito Santo in Brazil.[17] This database contains comprehensive COVID-19 records from year 2019 to year 2022. We used data collected during the years 2020, 2021, and 2022 for our analyses. Records with missing data fields were excluded from analysis. The final dataset for our study had records of 1,061,709 individuals. Figure 1 illustrates the entire workflow of the data preprocessing and model building for our analysis. Python (version 3.10.9) in conjunction with scikit-learn (version 1.0.2) were used in data preprocessing and model building.

### Model development

As illustrated in Fig. 1, we devised an algorithm leveraging a dataset with 20 distinct features. To ensure dataset balance, we first adjusted the data to have an equal number of positive and negative labels. The balanced dataset was then shuffled and subsequently divided into training (70%) and testing (30%) sets. Additionally, we assessed multicollinearity among the explanatory variables by calculating the Variance Inflation Factor (VIF) and Generalized Variance Inflation Factor (GVIF) for each feature. The highest VIF and GVIF values was for Age_range, but it was less than 10, indicating that multicollinearity is not a concern in our models. For specific VIF and GVIF values, see Appendix Table 1. To enhance model robustness and interpretability, we mainly used logistic regression (LR) [18–19] and random forest (RF) [20–21] to construct classification algorithms with regard to two different outcomes: COVID-19 infection and COVID-19 mortality. Model performance was evaluated based on Monte Carlo cross-validation (MCCV), [22] which is a cross-validation technique for assessing the performance of a machine learning model through multiple rounds of random data sampling and model training. It is also noteworthy that MCCV, in contrast to traditional K-fold cross-validation, generates diverse training and testing sets through repeated sampling and thus offers a more comprehensive model performance evaluation.

### Shapley values for machine learning

Most machine learning models are inherently non-linear, thus there is no straightforward way of evaluating the significance of individual features in machine learning models. Shapley values (SHapley Additive exPlanations, SHAP),[23] a game theory concept pioneered by economist Lloyd Shapley, allows us to evaluate the importance of each feature for a non-linear model. Shapley values quantify the marginal contribution of each feature towards the final output of the model. Shapley values treat each feature as an individual “player” within a cooperative “team” of features which collectively influence the predictions given by a machine learning model, a setting known as “coalitional game”. [24] In this paper, we compute Shapley values using the shap (version 0.41.0) package in Python.

To elaborate, the baseline output of a model is established by averaging of all its predictions. Subsequently, each individual prediction is analyzed as a function of feature influence, leading to deviations from the model’s baseline prediction. This concept of “influence” in making a positive or negative prediction is then rigorously examined through the use of various feature “teams,” which consist of different features. Through this approach, Shapley value provides a practical means of evaluating the impact of each feature on individual predictions by assessing its importance to the model’s output, both when considered in conjunction with other features and when analyzed in isolation. Shapley value also has unique theoretical properties: local accuracy, consistency and missingness, all of which ensure the robustness and interpretability of Shapley value.[23–27].

The Shapley value of each specific feature is computed as follows:

1 $$\begin{gathered}{\emptyset _k}(f) = {\Sigma _{s \subseteq \{ {x_1},...,{x_p}\} \backslash \{ {x_k}\} }} \hfill \\\frac{{\left| S \right|!(p - \left| S \right| - 1)!}}{{p!}}f(S \cup \{ {x_k}\} ) - f(s)) \hfill \\ \end{gathered}$$

$\:{\varnothing\:}_{k}\left(f\right)$ is the computed Shapley value for the feature k based on a prediction model f, and it quantifies the proportional contribution of feature k to the predicted outcomes of model f. According to Eq. (1), one needs to define the feature set and model, and then calculate the discrepancy in model predictions for each subset with and without feature k. For linear regression, the Shapley value of feature k is expressed as $\:{\varnothing\:}_{k}\left(\widehat{f}\right)={\beta\:}_{k}{x}_{k}-{\beta\:}_{k}E\left({X}_{k}\right)\:$to reflect the contribution of feature k to the prediction produced by the linear regression model. Furthermore, the sum of the Shapley values of individual features quantifies the overall contributions of all the model features regarding model predictions, i.e., $\:\sum\:_{k=1}^{p}{\varnothing\:}_{k}\left(\widehat{f}\right)=\widehat{f}\left(x\right)-E\left(\widehat{f}\right(X\left)\right)$.The sign of Shapley value indicates whether the feature positively or negatively influences the predicted value. By theory, Shapley value is an equitable and consistent estimate of the contribution of each model feature. [24–25] Moreover, Shapley value offers an interpretable approach for elucidating feature contributions to model predictions.

Considering a scenario where we have a machine learning model predicting COVID-19 mortality rates, the Shapley values for features such as age, hospitalization, and comorbidities are computed to quantify their respective contributions to the model’s predictions. If the Shapley value for age is 0.4, it indicates a positive contribution to the prediction, signifying that an increase in age correlates with a higher predicted mortality rate. In contrast, a negative Shapley value for comorbidities as -0.2 suggests a mitigating effect on the prediction, implying a reduced predicted mortality rate for individuals with comorbidities. Moreover, for this specific model, age has a greater impact than comorbidities on predictions, given the absolute Shapley value of age (0.4) is larger than the absolute Shapley value of comorbidities (0.2). This example illustrates how Shapley value help us understand individual feature contributions for complex machine learning models.

## Results

### Model for COVID-19 infection

Records of 1,061,709 individuals (57.06% male and 42.94% female) with features on demographic information, symptoms, comorbidities, health outcome and travel history were used for model training. Of all the participants, the positive rate was 28.55% across all years (32.68% in 2020, 26.15% in 2021, and 28.59% in 2022). The detailed descriptive statistics can be found in the Appendix Table 2. The model hyperparameters were determined via a combination of MCCV and grid search, and the performance evaluation metrics are provided by Table 1.

**Table 1:** A comparison of the model performances for predicting COVID-19 infections based on logistic regression (LR) and random forest (RF)

Model	Year (Test)	Year (Train)	Accuracy	ROC AUC	PR AUC	F1	Sensitivity	Specificity
LR	2020	2020	0.6053	0.6413	0.6304	0.5824	0.5544	0.6547
	2021	2021	0.5999	0.6340	0.6357	0.5813	0.5559	0.6437
		2020	0.5885	0.6206	0.6211	0.5788	0.5691	0.6064
	2022	2022	0.6295	0.6753	0.6524	0.6264	0.6169	0.6389
		2021	0.5870	0.6035	0.6055	0.5277	0.4657	0.7047
		2020	0.6065	0.6481	0.6305	0.5537	0.4924	0.7197
RF	2020	2020	0.5833	0.6037	0.5852	0.5767	0.5665	0.5930
	2021	2021	0.5710	0.5944	0.5822	0.5628	0.5507	0.5809
		2020	0.5537	0.5697	0.5577	0.5653	0.5822	0.5856
	2022	2022	0.6151	0.6504	0.6220	0.6187	0.6255	0.6320
		2021	0.5570	0.5700	0.5633	0.5188	0.4791	0.6327
		2020	0.5597	0.5763	0.5605	0.5474	0.5334	0.5874

In general, the metric values in Table 1 exhibit a pattern of temporal change in terms of model accuracies. With a testing set from year 2021, using a training set from the same year would bring an accuracy gain as 0.0114 (logistic regression) and 0.0173 (random forest) compared to using a training set from year 2020. With a testing set from year 2022, using a training set from the same year would bring accuracy gains as 0.0425 (logistic regression) and 0.0581 (random forest), compared to using a training set from year 2021. Compared to using a training set from year 2020, the accuracy gains are 0.023 (logistic regression) and 0.0554 (random forest) for using a training set from the same year as the testing set (i.e., year 2022). Similarly, gains in ROC AUC, PR AUC and F1 are also noticeable. The average gains for logistic regression are 0.0375, 0.0278 and 0.058 in ROC AUC, PR AUC and F1 score respectively. The average gains for random forest are 0.0597, 0.0482 and 0.0562 in ROC AUC, PR AUC and F1 score respectively. Those results align well with our earlier hypothesis, i.e., machine learning model typically underperforms when there is a temporal gap between the training set and the testing set [16]. We also observe that the performance gap is larger for testing set from year 2022 than testing set from year 2021, potentially attributed to the emergence of Omicron.

In addition, we observe that the sensitivities of both models (logistic regression and random forest) are generally quite low. The specificities of both models, although are still low, but are consistently higher than the sensitivities. This suggests that it is more difficult to claim someone as COVID-19 positive than ascertain he/she is negative, which aligns with our expectation. We also notice significant drops in sensitivity for both models when using a training set from year 2020 or 2021, compared to using a training set from year 2022 for a testing set from the same year. This may be due to the emergence of the Omicron variant whose symptoms are more similar to influenza rather than pneumonia, unlike most other previous SARS-CoV-2 variants.

**Fig. 2:**
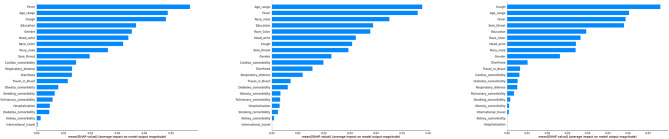
A comparison of the mean Shapley values of the features for random forest across three years of data (2020–2022). The left figure corresponds to the model using a training set and a testing set from year 2020. The middle figure corresponds to the model using a training set and a testing set from year 2021. The right figure corresponds to the model using a training set and a testing set from year 2022

To delve deeper into the investigation of temporal impact, we used Shapley values to quantify individual feature contributions. Figure 2 presents the mean Shapley values of notable model features for data from year 2020, 2021, and 2022 respectively, based on random forest. The top 5 (most important) model features for year 2020 are fever (yes/no), age, cough (yes/no), education and gender. The top 5 (most important) model features for year 2021 are age, fever, running nose (yes/no), education and race. The top 5 (most important) model features for year 2022 are cough (yes/no), age, fever, sore throat (yes/no) and education. It is clear that discrepancies exist among the feature rankings of the three years, which provide some insights into the model’s inconsistent predictive performance across the years. Although key predictors like age, fever and education appear in the top five list in every year, two features only show in the top five list in one year, namely running nose (yes/no) in year 2021 and sore throat (yes/no) in year 2022. In addition, cough did not make it into the top five list in year 2021. This suggest that different symptoms may exhibit at different stages of the pandemic, a known fact due to different SARS-CoV-2 variants that dominated the transmission dynamics at different stages of the pandemic. From Fig. 2, we also notice the lack of a fixed set of features that predominate the feature contributions towards prediction, which may destabilize model performance for predicting COVID-19 infections, particularly across different time periods.

### Model for COVID-19 mortality

Records of 298,292 individuals (55.41% male and 44.59% female) with features on demographic information, symptoms, comorbidities, health outcome and travel history were used for model training. Of all the participants, the mortality rate was 0.76% across all years (3.32% in 2020, 2.77% in 2021, and 0.19% in 2022). The detailed descriptive statistics can be found in the Appendix Table 3. The model hyperparameters were determined via a combination of MCCV and grid search, and the performance evaluation metrics are provided by Table 2.

**Table 2:** A comparison of the model performances for predicting COVID-19 mortality based on logistic regression (LR) and random forest (RF)

Model	Year (Test)	Year (Train)	Accuracy	ROC AUC	PR AUC	F1	Sensitivity	Specificity
LR	2020	2020	0.8901	0.9574	0.9540	0.8897	0.8790	0.9236
	2021	2021	0.8761	0.9486	0.9473	0.8747	0.8657	0.8706
		2020	0.8611	0.9451	0.9449	0.8523	0.7715	0.9351
	2022	2022	0.8888	0.9516	0.9586	0.8867	0.8539	0.9438
		2021	0.8758	0.9508	0.9561	0.8698	0.8107	0.9181
		2020	0.8736	0.9493	0.9589	0.8637	0.7938	0.9435
RF	2020	2020	0.8806	0.9519	0.9472	0.8804	0.8662	0.8790
	2021	2021	0.8665	0.9372	0.9352	0.8578	0.8259	0.8905
		2020	0.8638	0.937	0.9334	0.8573	0.8277	0.8951
	2022	2022	0.8781	0.9409	0.9428	0.8731	0.8539	0.9101
		2021	0.8640	0.9388	0.9414	0.8585	0.8164	0.9068
		2020	0.8653	0.9399	0.942	0.8571	0.8192	0.9181

In general, the metric values in Table 2 do not exhibit a strong pattern of temporal change. With a testing set from year 2021, using a training set from the same year would increase the accuracy by 0.015 (logistic regression) and 0.0027 (random forest) compared to using a training set from year 2020. With a test set from year 2022, using a training set from the same year would increase the accuracy by 0.013 (logistic regression) and 0.0141 (random forest), compared to using a training set from year 2021. Compared to using a training set from year 2020, the accuracy gains are 0.0152 (logistic regression) and 0.0128 (random forest) for using a training set from year 2022. Likewise, gains in ROC AUC, PR AUC and F1 are also inconsiderable. The average gains for logistic regression are 0.0022, 0.0015 and 0.0208 in ROC AUC, PR AUC and F1 score respectively. The average gains for random forest are 0.0011, 0.0013 and 0.0104 in ROC AUC, PR AUC and F1 score respectively. Those gains are much smaller than what we observed for predicting COVID-19 infection. Those results indicate there was little temporal impact on predicting COVID-19 mortality. In fact, the metric values are stably high across different years, indicating the ease of building machine models with high accuracy for COVID-19 mortality. In addition, we also observed the sensitivity was consistently lower than the specificity for COVID-19 mortality, although the sensitivity for predicting COVID-19 mortality was significantly higher than the sensitivity for predicting COVID-19 infection, suggesting that predicting COVID deaths is much less challenging than predicting COVID infections. This observation is consistent with the findings from previous research [16, 28].

**Fig. 3:**
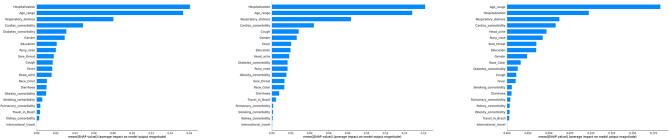
A comparison of the mean Shapley values of the features for random forest across three years of data (2020–2022). The left figure corresponds to the model using a training set and a testing set from year 2020. The middle figure corresponds to the model using a training set and a testing set from year 2021. The right figure corresponds to the model using a training set and a testing set from year 2022

To illustrate individual feature contributions, Fig. 3 presents the mean Shapley values of notable model features based on random forest with training and testing sets both from year 2020, 2021 and 2022 respectively. The top 5 (most important) model features for year 2020 are hospitalization (yes/no), age, respiratory distress (yes/no), cardiac comorbidity (yes/no) and diabetes comorbidity (yes/no). The top 5 (most important) model features for year 2021 are hospitalization (yes/no), age, respiratory distress (yes/no), cardiac comorbidity (yes/no) and cough (yes/no). The top 5 (most important) model features for year 2022 are age, hospitalization (yes/no), respiratory distress (yes/no), cardiac comorbidity (yes/no) and headache (yes/no). It is clear that the first four features of the top 5 list remain unchanged from 2020 to 2022, which largely explains the stability of model performance across the years. From Fig. 3, we also notice that the mean Shapley values of the first four features (i.e., age, hospitalization, respiratory distress and cardiac comorbidity) are much larger than the other features included in the model, indicating that the contributions of these four features dominate the total contributions towards prediction, and such dominance exists in all three years (2020–2022). In particular, age and hospitalization (yes/no) are the two most important features for predicting COVID-19 mortality for all three years. This key observation explains the high accuracy and stable performance of machine learning model for COVID-19 mortality prediction, as manifested in Table 2.

### Summary

We also make a general comparison between the random forest models for COVID-19 infection mortality, by pooling the data over 3 years. The infection model has a model accuracy as 60.47%, and its ROC AUC, PR AUC and F1 score are 63.82%, 61.21% and 60.67% respectively. Furthermore, it has a sensitivity of 61.11% and a specificity of 59.92%. Those metric values again evidence the poor performances of machine learning models for predicting COVID-19 infections, even with a large sample size. On the other hand, the mortality model has a model accuracy as 87.44%, and its ROC AUC, PR AUC and F1 score are 94.20%, 93.66% and 87.36% respectively. Moreover, it has a sensitivity of 86.13% and a specificity of 88.81%, demonstrating its strong predictive performance, particularly with a large volume of data.

**Fig. 4:**
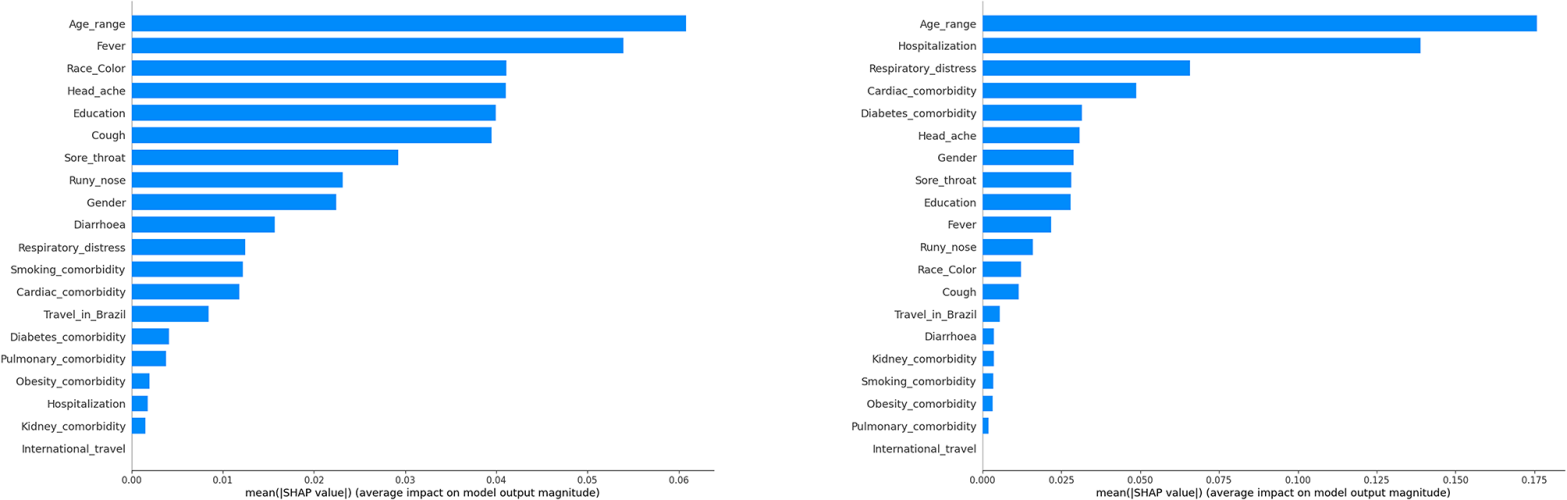
A comparison of the mean Shapley values of the features for random forest on three years data. The left figure corresponds to the model using a training set and a testing set by Infection model. The right figure corresponds to the model using a training set and a testing set by mortality model

Figure 4 presents the mean Shapley values of notable model features based on random forest, after combining all three years of data. The top 5 (most important) model features for the infection model are age, fever(yes/no), race, headache (yes/no) and education. The top 5 (most important) model features for the mortality model are age, hospitalization (yes/no), respiratory distress (yes/no), cardiac comorbidity (yes/no) and diabetes comorbidity (yes/no). Those feature lists are the same top features we obtained in the Sect. Model for COVID-19 infection and Model for COVID-19 mortality. More importantly, Fig. 4 reaffirms our earlier interpretations regarding Figs. 2 and 3 and i.e., the infection model has low accuracy and weak robustness across different years of data because there isn’t a fixed set of model features that dominate the contributions towards COVID-19 infection predictions. For comparison, the mortality model has much higher accuracy and stronger robustness across different years of data, as four model features (i.e., age, hospitalization, respiratory distress and cardiac comorbidity) dominate the contributions towards COVID-19 mortality predictions, and such dominance still exists for each single year of data from 2020 to 2022. Those four features, especially age and hospitalization, can largely predict COVID-19 mortality even for different periods (SARS-CoV-2 variants) during the pandemic, which essentially leads to the strong robustness of the mortality model.

To further validate our findings, we conducted additional analyses using SHAP on XGBoost models and Permutation Importance on random forest models (Appendix Fig. 1 ~ 4). The results aligned well with our earlier observations. Specifically, the SHAP values from XGBoost models confirmed that the infection model lacks a stable set of dominant features across different years, while the mortality model had the same top four features (age, hospitalization, respiratory distress, and cardiac comorbidity) as the most significant predictors across the years. Similarly, Permutation Importance applied to the random forest models yielded identical feature importance rankings, reinforcing the robustness of our conclusions. These results across different interpretability indices and models highlight the reliability of our findings and underscore the temporal stability of the mortality model compared to the infection model.

## Discussion

This study investigates the influence of temporality on machine learning models for predicting COVID-19 infection and mortality. By analyzing an extensive COVID-19 dataset spanning three years during the pandemic (2020–2022) from Brazil, this study explores how the performances of logistic regression (LR) and random forest (RF) models are affected by the temporal gap between the training set and the testing set, i.e., if the training and testing sets are from different years. The results indicate that there was likely a loss in accuracy for predicting COVID-19 infection (both LR and RF included) when the temporal gap between the training and testing sets existed. On the contrary, we found no significant accuracy loss for predicting COVID-19 mortality (both LR and RF included) given the temporal gap between the training and testing sets. To deepen the understanding of the above temporal impact, Shapley values were employed to quantify the contribution of each model feature towards predictions. Shapley values revealed that the set of features that made most contribution towards COVID-19 infection prediction was not fixed and potentially varied across different years. In other words, there was lack of a fixed set of model features that dominate the contributions towards COVID-19 infection prediction across three years, and consequently machine learning models for predicting COVID-19 infections generally had low accuracy and were further undermined by the temporal gap between the training and testing sets. On the other hand, via Shapley values, we found there were four features that dominated the contributions towards COVID-19 mortality prediction across three years, namely, age, hospitalization (yes/no), respiratory distress (yes/no) and cardiac comorbidity (yes/no). Particularly, age and hospitalization (yes/no) made much more contributions than all other features across three years, which clearly confirmed the existence of a fixed set of features that dominated the contributions towards COVID-19 mortality prediction, a phenomenon that did not appear for COVID-19 infection prediction. As a result, machine learning models for predicting COVID-19 mortality had much higher accuracy and stronger robustness against the temporal gap between the training and testing sets.

Shapley value offers an enlightening perspective of predictability and model robustness, as it can quantify individual feature importance in a fairly interpretable and comparative way. For predicting COVID-19 infection, Shapley value suggested that feature importance may vary across different years of data, i.e., there was no fixed set of features that dominated the contributions towards infection prediction. Specifically, we found runny nose as a new important feature emerging from year 2021 and sore throat as a new important feature emerging from year 2022, which implies that COVID-19 infections may have different symptoms at different years, a clinical observation that aligns with most literature.[29–33] In addition, we also found the symptom “fever” became less important during 2020–2022, mostly due to clinical features of different COVID variants. This largely explains why the infection model had relatively low accuracy and weak robustness regarding temporality. In contrast, for predicting COVID-19 mortality, Shapley value helped identify the same four features (age, hospitalization, respiratory distress and cardiac comorbidity) that dominated the contributions towards predictions across the three years (2020–2022). Clinically, those four features all have strong relationships with COVID-19 mortality. For example, heart disease is a well-established risk factor associated with an increased risk of death in COVID-19 patients. [34–36] Autopsy results using RT-PCR revealed the presence of viruses in cardiac tissue and myocarditis was associated with elevated cardiac biomarkers. Additionally, documented cases of cardiac arrhythmias in COVID-19 patients also add to the overall complexity. [37] This provides a reasonable explanation for the observed increased risk of death in patients with heart disease. Age, [38 hospitalization^39^ and respiratory distress^40^–^41^] are also confirmed by literature to be strong predictors of COVID-19 mortality. This provides convincing reasons of why the mortality model is more robust and accurate than the infection model, and it demonstrates the high interpretability of Shapley values, both mathematically and clinically. To strengthen the robustness of our findings, we also cross-validated feature importance rankings using both Shapley values and permutation importance index across infection and mortality prediction models (Appendix Figs. 3 and 4). Notably, both indices yielded consistent conclusions: the infection model exhibited temporal instability in predictive features, while the mortality model was significantly more stable due to the dominance of the same four features (age, hospitalization, respiratory distress, and cardiac comorbidity) across all years. Minor discrepancies, e.g. “Fever” was prioritized by SHAP while “Education” and “Race Color” were prioritized by permutation importance, highlight the complementary strengths of these two indices. Specifically, SHAP provides granular, instance-level insights sensitive to feature interactions, and permutation importance quantifies global feature impacts through performance degradation. Therefore, it’s important to verify the consistency of machine learning results by comparing multiple interpretability indices to ensure robust, clinically actionable insights.

The scholarly significance of this study is threefold: First, by placing training and testing data at different years, our study discovered that temporality had a negative impact on COVID-19 infection predictions. That is, a gap between training and testing sets would likely undermine model accuracy and robustness for predicting COVID-19 infections. However, we did not find such negative impact of temporality for predicting COVID-19 mortality, given the gap between the training and testing sets. Second, our study offers a new perspective of model robustness investigation, i.e., through Shapley value. Shapley value evaluates individual feature contributions towards predictions, and therefore it enables us to compare feature importance and identify the underlying set of features that drive the predictions. Shapley value uncovers that model robustness depends on the existence of a fixed set of features that make predominant contributions to model predictions. For our investigation, the infection model did not have such fixed set of features while the mortality model did have a fixed set of four features, for datasets from different years. Shapley value imparts key insights regarding the driving forces behind model robustness. Third, our findings have important empirical implications for improving the accuracy and robustness of machine learning models built for COVID-19 related predictions, i.e., one should pay close attention to the potential temporal gap between the training and testing sets and be cautious when using/interpreting a machine learning model trained with old data. For modelers, it is advisable to use Shapley values to quantify individual feature importance such that users can gain deeper insights as to why a machine learning model works (or does not work well). It our hope that, via a more careful model robustness analyses using Shapley values, public awareness about the stability and interpretability of machine learning models can be raised, which likely facilitates the planning of effective interventions and clinical diagnoses in a future public health crisis.

## Limitation

Our study does have limitations: First, our study relies only on COVID-19 data from the Brazilian state of Espírito Santo. Generalizability may be limited by differences in demographics, health care infrastructure, and population behavior across countries. Caution is required when extrapolating these findings to other countries. This limitation may affect the generalizability of our conclusions, indicating that the observed patterns and model performances might not be directly applicable to other settings with different characteristics. Second, we did not take the transmission dynamics of SARS-CoV-2 into account. While the study focuses on the temporality which is largely implicated by the occurrences of distinct SARS-CoV-2 variants, it does not investigate the impact of specific variants on model performance. It’s notable that the impact of temporality could be confounded by possible temporal overlaps between different variants, [42] vaccination effects, [43–44] and other temporal changes, and thus it is more complex than we estimated in this paper. The study also might omit some important features that could affect model performance, such as vaccination rates, public health policies or socioeconomic statuses. [45] These omitted features may significantly influence the transmission dynamics of SARS-CoV-2 and thus bias our estimates. Because of these limitations, our findings should be interpreted with caution in an epidemiological context. For this reason, some literature suggests that using epidemiological models may be helpful and that using machine learning models alone may not be sufficient. [46, 47, 48] Third, we mainly considered logistic regression (LR) and random forest (RF) in this paper, as both LR and RF have high interpretability and simplicity, which makes LR and RF more stable and potentially better choice for using Shapley values. However, this limitation may mean that our conclusions about model performance and feature importance might differ if more complex models were employed, potentially affecting the generalizability of our findings to other modeling approaches. Future research should explore methods to adapt models to overcome temporal gaps using techniques such as transfer learning, dynamic updating, or time-series analysis. These approaches could help address the temporal dynamics and improve model performance across different time periods. By incorporating these advanced techniques, future studies may enhance the robustness and generalizability of predictive models for infectious diseases like COVID-19. Fourth, different data sampling strategies can also affect model performance and interpretation. In this study, we adopted a random undersampling approach to preserve the real-world data distribution, reduce computational overhead, and mitigate overfitting risks. However, oversampling methods such as SMOTE represent viable alternatives that might yield different outcomes. Future work could conduct a comparison study of various sampling methods to further examine the impact of data preprocessing on our findings. Fifth, this study focuses on annual time windows, thus overlooking shorter-term fluctuations that could provide more immediate insights into COVID-19 transmission and disease progression. While this approach allows for a clearer examination of broader temporal effects, weekly or monthly analyses may reveal additional nuances in model performance and feature importance. Future work should incorporate these finer temporal resolutions to further enhance COVID related predictions.

## Conclusion

In conclusion, our study confirmed the impact of temporality on model performances for predicting COVID-19 infection, but we did not find such impact for predicting COVID-19 mortality. Specifically, a temporal gap between the training and testing sets would reduce the model accuracy for predicting COVID-19 infection, but such gap had little impact on the model accuracy for predicting COVID-19 mortality. A follow-up analysis based on Shapley values uncovered the reasons behind this observation: a fixed set of four features (i.e., age, hospitalization (yes/no), respiratory distress (yes/no) and cardiac comorbidity (yes/no)) were the main contributors for predicting COVID-19 mortality across three years (2020–2022), however, for predicting COVID-19 infection, the main contributing features were not fixed across three years, which was potentially attributed to the distinctions among SARS-CoV-2 variants during that time. Our study shows that results from machine learning models should be interpreted with caution as temporality and possibly other confounders can reduce model accuracy. Our study also demonstrates that model robustness is connected to the stability of feature contributions, i.e., whether a fixed set of features can significantly contribute to model predictions across various scenarios. These findings hold practical significance for decision-makers, as they highlight the importance of considering temporal factors and feature stability when developing predictive models for public health interventions.

## Electronic supplementary material

Below is the link to the electronic supplementary material.

Supplementary Material 1

## Data Availability

No datasets were generated or analysed during the current study.
