# Supplementary material for: An investigation into the impact of temporality on COVID-19 infection and mortality predictions: new perspective based on Shapley Values
Source: BMC Med Res Methodol. 2025 Apr 24;25:111. doi: 10.1186/s12874-025-02572-8 (PMC12020040; doi:10.1186/s12874-025-02572-8)

**Appendix**

Appendix Table 1: Variance Inflation Factor (VIF) and Generalized Variance Inflation Factor (GVIF) Values for Parameters in COVID-19 Infection and Mortality Models

| Feature | VIF | | GVIF | |
| --- | --- | --- | --- | --- |
|  | infection model | mortality model | infection model | mortality model |
| Age_range | 6.846622 | 7.492763 | 2.616605 | 2.737291 |
| Gender | 1.621553 | 1.643516 | 1.273402 | 1.281997 |
| Race_Color | 4.744245 | 4.861075 | 2.178129 | 2.204785 |
| Education | 4.558514 | 4.604515 | 2.135068 | 2.145813 |
| Fever | 1.514786 | 1.727422 | 1.230766 | 1.314314 |
| Respiratory_distress | 1.148900 | 1.161617 | 1.071868 | 1.077783 |
| Cough | 2.233122 | 2.624284 | 1.494363 | 1.619964 |
| Runy_nose | 1.886843 | 1.982485 | 1.373624 | 1.408007 |
| Sore_throat | 1.798693 | 1.906094 | 1.341154 | 1.380614 |
| Diarrhoea | 1.119435 | 1.120217 | 1.058034 | 1.058403 |
| Head_ache | 1.969106 | 2.150286 | 1.403248 | 1.466385 |
| Pulmonary_comorbidity | 1.034508 | 1.026828 | 1.017108 | 1.013325 |
| Cardiac_comorbidity | 1.369307 | 1.396622 | 1.170174 | 1.181788 |
| Kidney_comorbidity | 1.014921 | 1.014128 | 1.007433 | 1.007039 |
| Diabetes_comorbidity | 1.200266 | 1.206503 | 1.095567 | 1.098409 |
| Smoking_comorbidity | 1.025040 | 1.020199 | 1.012443 | 1.010049 |
| Obesity_comorbidity | 1.035881 | 1.044843 | 1.017782 | 1.022176 |
| Hospitalization | 1.044183 | 1.075806 | 1.021853 | 1.037211 |
| Travel_in_Brazil | 1.051470 | 1.058118 | 1.025412 | 1.028649 |
| International_travel | 1.004073 | 1.004052 | 1.002034 | 1.002024 |

Appendix Table 2: Demographic data and clinical characteristics of the study for infection model

|  | Negative n (%) | Positive n (%) | All n (%) |
| --- | --- | --- | --- |
| Confirmation Criteria |  |  |  |
| Confirmation | 731531 (95.82) | 297814 (99.84) | 1029345 (96.95) |
| Unknown | 31886 (4.18) | 478 (0.16) | 32364 (3.05) |
| Age range |  |  |  |
| 0-4 years old | 3586 (0.47) | 933 (0.31) | 4519 (0.43) |
| 5-9 years old | 25851 (3.39) | 6873 (2.3) | 32724 (3.08) |
| 10-19 years old | 87287 (11.43) | 24896 (8.35) | 112183 (10.57) |
| 20-29 years old | 148614 (19.47) | 49681 (16.66) | 198295 (18.68) |
| 30-39 years old | 160735 (21.05) | 62580 (20.98) | 223315 (21.03) |
| 40-49 years old | 137379 (18.0) | 58217 (19.52) | 195596 (18.42) |
| 50-59 years old | 97910 (12.83) | 44619 (14.96) | 142529 (13.42) |
| 60-69 years old | 62265 (8.16) | 29912 (10.03) | 92177 (8.68) |
| 70-79 years old | 27164 (3.56) | 14099 (4.73) | 41263 (3.89) |
| 80-89 years old | 10401 (1.36) | 5363 (1.8) | 15764 (1.48) |
| 90 years old or more | 2222 (0.29) | 1116 (0.37) | 3338 (0.31) |
| Unknown | 3 (0.0) | 3 (0.0) | 6 (0.0) |
| Gender |  |  |  |
| Male | 307610 (40.29) | 121834 (40.84) | 429444 (40.45) |
| Female | 455807 (59.71) | 176458 (59.16) | 632265 (59.55) |
| Race/Color |  |  |  |
| Asian | 40917 (5.36) | 16388 (5.49) | 57305 (5.4) |
| White | 268987 (35.23) | 116153 (38.94) | 385140 (36.28) |
| Black | 48126 (6.3) | 16692 (5.6) | 64818 (6.11) |
| Indigenous | 581 (0.08) | 212 (0.07) | 793 (0.07) |
| Brown | 318917 (41.77) | 115092 (38.58) | 434009 (40.88) |
| Unknown | 85889 (11.25) | 33755 (11.32) | 119644 (11.27) |
| Education |  |  |  |
| iES (1-4 grade) | 53731 (7.04) | 21326 (7.15) | 75057 (7.07) |
| cES (4 grade) | 31733 (4.16) | 14818 (4.97) | 46551 (4.38) |
| iES (5-8 grade) | 86904 (11.38) | 33328 (11.17) | 120232 (11.32) |
| cES (8 grade) | 55219 (7.23) | 23213 (7.78) | 78432 (7.39) |
| iHS | 63355 (8.3) | 22800 (7.64) | 86155 (8.11) |
| cHS | 245444 (32.15) | 99308 (33.29) | 344752 (32.47) |
| iHE | 38828 (5.09) | 12747 (4.27) | 51575 (4.86) |
| cHE | 175846 (23.03) | 65740 (22.04) | 241586 (22.75) |
| llliterate | 12357 (1.62) | 5012 (1.68) | 17369 (1.64) |
| Hospitalization |  |  |  |
| Yes | 2225 (0.29) | 2195 (0.74) | 4420 (0.42) |
| No | 761192 (99.71) | 296097 (99.26) | 1057289 (99.58) |
| Brazil Travel |  |  |  |
| Yes | 25673 (3.36) | 12780 (4.28) | 38453 (3.62) |
| No | 737744 (96.64) | 285512 (95.72) | 1023256 (96.38) |
| International Travel |  |  |  |
| Yes | 716 (0.09) | 220 (0.07) | 936 (0.09) |
| No | 762701 (99.91) | 298072 (99.93) | 1060773 (99.91) |
| Fever |  |  |  |
| Yes | 162481 (21.28) | 115477 (38.71) | 277958 (26.18) |
| No | 600936 (78.72) | 182815 (61.29) | 783751 (73.82) |
| Respiratory Distress |  |  |  |
| Yes | 53938 (7.07) | 24482 (8.21) | 78420 (7.39) |
| No | 709479 (92.93) | 273810 (91.79) | 983289 (92.61) |
| Cough |  |  |  |
| Yes | 309921 (40.6) | 172906 (57.97) | 482827 (45.48) |
| No | 453496 (59.4) | 125386 (42.03) | 578882 (54.52) |
| Runny nose |  |  |  |
| Yes | 267803 (35.08) | 133300 (44.69) | 401103 (37.78) |
| No | 495614 (64.92) | 164992 (55.31) | 660606 (62.22) |
| Sore Throat |  |  |  |
| Yes | 255334 (33.45) | 133300 (44.69) | 388634 (36.6) |
| No | 508083 (66.55) | 164992 (55.31) | 673075 (63.4) |
| Diarrhea |  |  |  |
| Yes | 54798 (7.18) | 24301 (8.15) | 79099 (7.45) |
| No | 708619 (92.82) | 273991 (91.85) | 982610 (92.55) |
| Headache |  |  |  |
| Yes | 286379 (37.51) | 150490 (50.45) | 436869 (41.15) |
| No | 477038 (62.49) | 147802 (49.55) | 624840 (58.85) |
| Pulmonary comorbidity |  |  |  |
| Yes | 14558 (1.91) | 4675 (1.57) | 19233 (1.81) |
| No | 748859 (98.09) | 293617 (98.43) | 1042476 (98.19) |
| Cardiac comorbidity |  |  |  |
| Yes | 65601 (8.59) | 32930 (11.04) | 98531 (9.28) |
| No | 697816 (91.41) | 265362 (88.96) | 963178 (90.72) |
| Kidney comorbidity |  |  |  |
| Yes | 1669 (0.22) | 751 (0.25) | 2420 (0.23) |
| No | 761748 (99.78) | 297541 (99.75) | 1059289 (99.77) |
| Diabetes comorbidity |  |  |  |
| Yes | 22320 (2.92) | 11094 (3.72) | 33414 (3.15) |
| No | 741097 (97.08) | 287198 (96.28) | 1028295 (96.85) |
| Smoking comorbidity |  |  |  |
| Yes | 10217 (1.34) | 2950 (0.99) | 13167 (1.24) |
| No | 753200 (98.66) | 295342 (99.01) | 1048542 (98.76) |
| Obesity comorbidity |  |  |  |
| Yes | 7949 (1.04) | 4043 (1.36) | 11992 (1.13) |
| No | 755468 (98.96) | 294249 (98.64) | 1049717 (98.87) |

Appendix Table 3: Demographic data and clinical characteristics of the study for mortality model

|  | Cure n (%) | Death n (%) | All n (%) |
| --- | --- | --- | --- |
| Confirmation Criteria |  |  |  |
| Confirmation | 295588 (99.84) | 2226 (99.55) | 297814 (99.84) |
| Unknown | 468 (0.16) | 10 (0.45) | 478 (0.16) |
| Age range |  |  |  |
| 0-4 years old | 933 (0.32) | 0 (0.0) | 933 (0.31) |
| 5-9 years old | 6871 (2.32) | 2 (0.09) | 6873 (2.3) |
| 10-19 years old | 24894 (8.41) | 2 (0.09) | 24896 (8.35) |
| 20-29 years old | 49649 (16.77) | 32 (1.43) | 49681 (16.66) |
| 30-39 years old | 62498 (21.11) | 82 (3.67) | 62580 (20.98) |
| 40-49 years old | 58042 (19.61) | 175 (7.83) | 58217 (19.52) |
| 50-59 years old | 44296 (14.96) | 323 (14.45) | 44619 (14.96) |
| 60-69 years old | 29400 (9.93) | 512 (22.9) | 29912 (10.03) |
| 70-79 years old | 13559 (4.58) | 540 (24.15) | 14099 (4.73) |
| 80-89 years old | 4957 (1.67) | 406 (18.16) | 5363 (1.8) |
| 90 years old or more | 954 (0.32) | 162 (7.25) | 1116 (0.37) |
| Unknown | 3 (0.0) | 0 (0.0) | 3 (0.0) |
| Gender |  |  |  |
| Male | 120617 (40.74) | 1217 (54.43) | 121834 (40.84) |
| Female | 175439 (59.26) | 1019 (45.57) | 176458 (59.16) |
| Race/Color |  |  |  |
| Asian | 16188 (5.47) | 200 (8.94) | 16388 (5.49) |
| White | 115283 (38.94) | 870 (38.91) | 116153 (38.94) |
| Black | 16479 (5.57) | 213 (9.53) | 16692 (5.6) |
| Indigenous | 208 (0.07) | 4 (0.18) | 212 (0.07) |
| Brown | 114255 (38.59) | 837 (37.43) | 115092 (38.58) |
| Unknown | 33643 (11.36) | 112 (5.01) | 33755 (11.32) |
| Education |  |  |  |
| iES (1-4 grade) | 20810 (7.03) | 516 (23.08) | 21326 (7.15) |
| cES (4 grade) | 14566 (4.92) | 252 (11.27) | 14818 (4.97) |
| iES (5-8 grade) | 33004 (11.15) | 324 (14.49) | 33328 (11.17) |
| cES (8 grade) | 22956 (7.75) | 257 (11.49) | 23213 (7.78) |
| iHS | 22706 (7.67) | 94 (4.2) | 22800 (7.64) |
| cHS | 98933 (33.42) | 375 (16.77) | 99308 (33.29) |
| iHE | 12729 (4.3) | 18 (0.81) | 12747 (4.27) |
| cHE | 65609 (22.16) | 131 (5.86) | 65740 (22.04) |
| llliterate | 4743 (1.6) | 269 (12.03) | 5012 (1.68) |
| Hospitalization |  |  |  |
| Yes | 1050 (0.35) | 1145 (51.21) | 2195 (0.74) |
| No | 295006 (99.65) | 1091 (48.79) | 296097 (99.26) |
| Brazil Travel |  |  |  |
| Yes | 12697 (4.29) | 83 (3.71) | 12780 (4.28) |
| No | 283359 (95.71) | 2153 (96.29) | 285512 (95.72) |
| International Travel |  |  |  |
| Yes | 220 (0.07) | 0 (0.0) | 220 (0.07) |
| No | 295836 (99.93) | 2236 (100.0) | 298072 (99.93) |
| Fever |  |  |  |
| Yes | 114286 (38.6) | 1191 (53.26) | 115477 (38.71) |
| No | 181770 (61.4) | 1045 (46.74) | 182815 (61.29) |
| Respiratory Distress |  |  |  |
| Yes | 23182 (7.83) | 1300 (58.14) | 24482 (8.21) |
| No | 272874 (92.17) | 936 (41.86) | 273810 (91.79) |
| Cough |  |  |  |
| Yes | 171399 (57.89) | 1507 (67.4) | 172906 (57.97) |
| No | 124657 (42.11) | 729 (32.6) | 125386 (42.03) |
| Runny nose |  |  |  |
| Yes | 137290 (46.37) | 513 (22.94) | 137803 (46.2) |
| No | 158766 (53.63) | 1723 (77.06) | 160489 (53.8) |
| Sore Throat |  |  |  |
| Yes | 132913 (44.89) | 387 (17.31) | 133300 (44.69) |
| No | 163143 (55.11) | 1849 (82.69) | 164992 (55.31) |
| Diarrhea |  |  |  |
| Yes | 24009 (8.11) | 292 (13.06) | 24301 (8.15) |
| No | 272047 (91.89) | 1944 (86.94) | 273991 (91.85) |
| Headache |  |  |  |
| Yes | 149792 (50.6) | 698 (31.22) | 150490 (50.45) |
| No | 146264 (49.4) | 1538 (68.78) | 147802 (49.55) |
| Pulmonary comorbidity |  |  |  |
| Yes | 4501 (1.52) | 174 (7.78) | 4675 (1.57) |
| No | 291555 (98.48) | 2062 (92.22) | 293617 (98.43) |
| Cardiac comorbidity |  |  |  |
| Yes | 31659 (10.69) | 1271 (56.84) | 32930 (11.04) |
| No | 264397 (89.31) | 965 (43.16) | 265362 (88.96) |
| Kidney comorbidity |  |  |  |
| Yes | 646 (0.22) | 105 (4.7) | 751 (0.25) |
| No | 295410 (99.78) | 2131 (95.3) | 297541 (99.75) |
| Diabetes comorbidity |  |  |  |
| Yes | 10428 (3.52) | 666 (29.79) | 11094 (3.72) |
| No | 285628 (96.48) | 1570 (70.21) | 287198 (96.28) |
| Smoking comorbidity |  |  |  |
| Yes | 2817 (0.95) | 133 (5.95) | 2950 (0.99) |
| No | 293239 (99.05) | 2103 (94.05) | 295342 (99.01) |
| Obesity comorbidity |  |  |  |
| Yes | 3754 (1.27) | 289 (12.92) | 4043 (1.36) |
| No | 292302 (98.73) | 1947 (87.08) | 294249 (98.64) |

Appendix Figure 1: A comparison of the mean Shapley values of the features for predicting COVID-19 infections using XGBoost across three years of data (2020-2022). The left panel displays results from the model using 2020 training and testing sets, the middle panel shows 2021 sets, and the right panel presents 2022 sets.


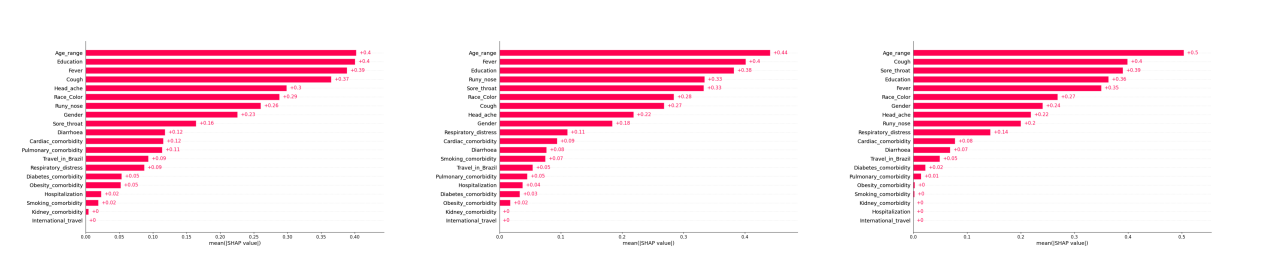


Appendix Figure 2: A comparison of the mean Shapley values of the features for predicting COVID-19 mortality using XGBoost across three years of data (2020-2022). The left panel displays results from the model using 2020 training and testing sets, the middle panel shows 2021 sets, and the right panel presents 2022 sets.


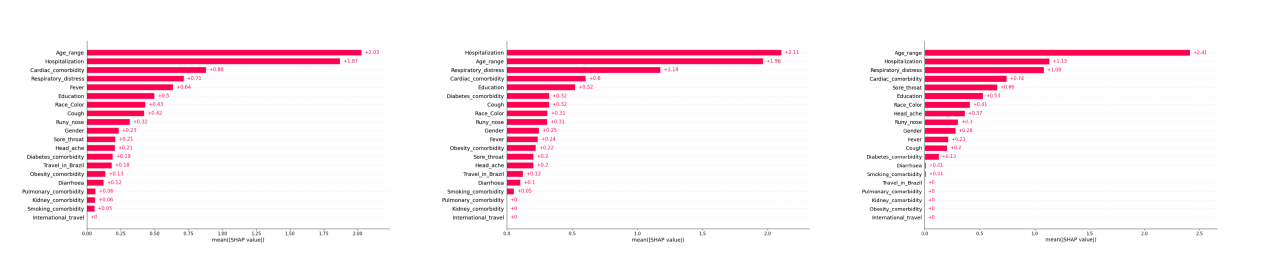


Appendix Figure 3: A comparison of the permutation importance values of the features for predicting COVID-19 infections using random forest across three years of data (2020-2022). The left panel displays results from the model using 2020 training and testing sets, the middle panel shows 2021 sets, and the right panel presents 2022 sets.

*
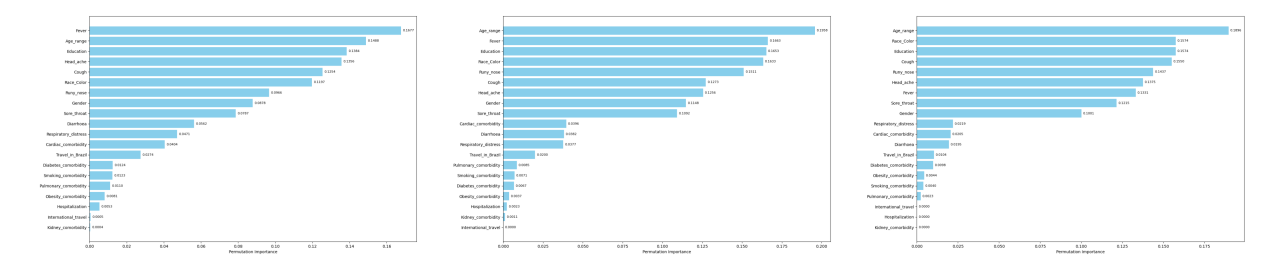
*

Appendix Figure 4: A comparison of the permutation importance values of the features for predicting COVID-19 mortality using random forest across three years of data (2020-2022). The left panel displays results from the model using 2020 training and testing sets, the middle panel shows 2021 sets, and the right panel presents 2022 sets.


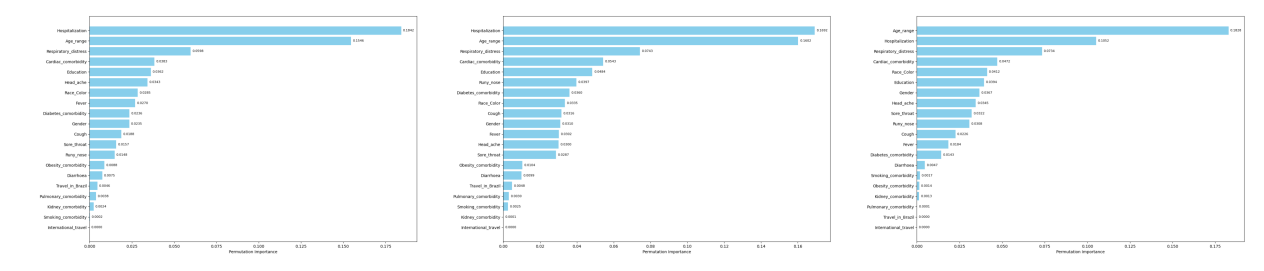

Supplement: Supplementary file 1 — Supplementary Material 1 [file 12874_2025_2572_MOESM1_ESM.docx]
